# Allogeneic Hematopoietic Cell Transplant for Systemic Juvenile Idiopathic Arthritis and Macrophage Activation Syndrome

**DOI:** 10.1155/2021/9323141

**Published:** 2021-05-24

**Authors:** Nicole Davidson, Hemalatha G. Rangarajan, Kyla Driest, Rajinder P. S. Bajwa, Veronika Polishchuk, Rolla F. Abu-Arja

**Affiliations:** ^1^Pediatric Residency Program, Nationwide Children's Hospital, Columbus, OH, USA; ^2^Pediatric Hematology, Oncology, Blood and Marrow Transplant, Nationwide Children's Hospital, Columbus, OH, USA; ^3^Department of Pediatrics, The Ohio State University, OH, USA; ^4^Pediatric Rheumatology, Nationwide Children's Hospital, Columbus, OH, USA

## Abstract

Systemic juvenile idiopathic arthritis (sJIA) is characterized by arthritis, fever, rash, lymphadenopathy, hepatosplenomegaly, and serositis. Macrophage activation syndrome is the most feared complication of sJIA with a high risk of mortality. We report a 16-year-old female diagnosed with refractory systemic juvenile idiopathic arthritis (sJIA) complicated by recurrent macrophage activation syndrome (MAS), severe joint disease, and lung involvement requiring prolonged immunosuppressive therapy. She received a matched unrelated allogeneic hematopoietic cell transplant (Allo-HCT) using a reduced-intensity conditioning regimen and is now, 3 years after the transplant, with complete resolution of sJIA symptoms, off immunosuppressants, and with significant improvement in the quality of life.

## 1. Introduction

Juvenile idiopathic arthritis (JIA) is the most common childhood chronic rheumatologic disorder and used to describe several forms of arthritis that develop in children under age 16 [1]. Systemic JIA (sJIA) is characterized by a combination of arthritis and systemic inflammatory symptoms including fever, rash, lymphadenopathy, hepatomegaly/splenomegaly, and/or serositis [2]. A life-threatening complication (mortality rate: 8%–17%) of sJIA is macrophage activation syndrome (MAS) [3]. MAS is a form of secondary hemophagocytic lymphohistiocytosis (HLH) that manifests with overproduction of cytokines and a hyperinflammatory state associated with cytopenia, liver dysfunction, and coagulopathy, resembling disseminated intravascular coagulation and, in severe cases, leading to organ failure [4, 5]. Therapy includes nonsteroidal anti-inflammatory drugs, long-term use of corticosteroids, calcineurin inhibitors, intravenous immunoglobulin, and other biologic agents, with anakinra being the most selected agent [6, 7]. Here, we describe a female who is now 22 years old with refractory sJIA and recurrent MAS who received a matched unrelated allogeneic hematopoietic cell transplant (Allo-HCT).

## 2. Case Description

The female patient was diagnosed with sJIA at age 16. She presented with intermittent fevers, fatigue, skin rash, and severe polyarthritis. At age 18, she developed the first episode of MAS and subsequently had multiple flares requiring prolonged hospitalizations and intensive care unit admissions. Over a course of 3 years, she was treated with multiple immunosuppressive therapies including adalimumab, tocilizumab, prednisone, intravenous immunoglobulin, anakinra, canakinumab, leflunomide, methotrexate, cyclosporine, and tacrolimus. She was then maintained on prednisone (1 mg/kg/day), tacrolimus, anakinra, hydroxychloroquine, and monthly intravenous immunoglobulins. Her workup included an HLH gene panel that showed a heterozygous variant of unknown significance (c. 1022G > A(p.Arg341His)) in AP3B1.

Due to her history of the recurrent MAS flares, lung involvement manifesting with abnormal DLCO, and the severity of joint disease along with the need for prolonged immunosuppressive therapy that severely impacted her quality of life, the decision was made to proceed with Allo-HCT using the best available matched unrelated donor (MUD).

A 10/10 HLA-matched unrelated male donor was identified through the National Marrow Donor Program. The patient received a reduced-intensity conditioning (RIC) with intermediate alemtuzumab 0.2 mg/kg per day (days (D)-14 to -12) followed by fludarabine 30 mg/m^2^ per day (D-8 to -4) and melphalan 140 mg/m^2^ (D-2). Her graft-versus-host disease (GvHD) prophylaxis included tacrolimus and methylprednisone. Hydroxychloroquine was discontinued before the start of conditioning, and daily anakinra was discontinued after engraftment. She received an unprocessed, allogeneic bone marrow for the stem cell source (9.75 × 10e6 CD34/kg and 0.78 × 10e8 CD3/kg) on D0. She attained neutrophil engraftment on D+9 and platelet engraftment on D+22 and was discharged on D+14. Peripheral blood chimerism on D+30, D+60, D+100, 1 year, and 2 years after HCT showed 99–100% donor chimerism in all fractions.

Early transplant-related complications included grade 2 mucositis, febrile neutropenia, steroid-induced hyperglycemia, grade 1 GvHD (skin stage I), and EBV reactivation followed by temporary B-cell aplasia secondary to rituximab therapy. Given her pre-HCT history of prolonged corticosteroid therapy for 3 years, she developed secondary adrenal insufficiency (AI) which necessitated a prolonged prednisone taper that was transitioned to physiological hydrocortisone dosing at 18 months after HCT. Rituximab along with prolonged corticosteroid therapy leads to delayed immune reconstitution that was associated with recurrent sinopulmonary infections.

The patient is now, 3 years after HCT, with full immune reconstitution and off prophylactic medications with no evidence of GVHD. She reports full movement and flexibility in all joints with no symptoms of sJIA.

## 3. Discussion

Patients with sJIA can experience significant morbidity associated with poorly controlled disease, long-term immunosuppressive therapy, serious infections, and autoinflammation; MAS is a known complication of sJIA which can lead to multiorgan system failure and death [7]. In a case series of 362 patients with sJIA-associated MAS, approximately 1/3 of patients required ICU-level care with an 8% mortality rate [6]. Our patient was treated with a prolonged course of systemic corticosteroids with a combination of immunosuppressive therapies for 3 years. She was frequently hospitalized with life-threatening recurrent MAS and was susceptible to infections. In addition, she developed severe joint disease that significantly affected her mobility and quality of life.

The use of HCT, by “resetting” the defective immune system, can offer a curative option for patients with life-threatening or debilitating sJIA. Case reports of sJIA treated with both autologous (auto) and allo-HCT have shown varying degrees of long-term success. Complete remission (CR) of the disease was observed in >50% of auto and >75% of allo-HCT recipients [8–11].

Much of the literature surrounding HCT in sJIA focuses on the use of auto-HCT as a curative option. In a study of 7 pediatric auto-HCT recipients, 4 achieved remission, 2 relapsed (1 within weeks and 1 within a year), and 1 died due to disseminated adenovirus [9]. One of the relapses occurred with an episode of MAS [9]. Similarly, in a larger study of 34 pediatric auto-HCT recipients, 53% achieved CR, 18% were partial responders (PRs), and 21% showed no response (NR) [10]. In a subsequent follow-up of 22 children in this cohort, 8 out of 20 evaluable patients remained in CR, 7 were PRs, and 5 relapsed. Of those who relapsed, 4 died: 2 related to infections after restarting immunosuppressive therapy and 2 because of recurrent MAS after transplant [11].

Silva et al. analyzed the outcomes of 16 patients with JIA who underwent allogeneic HCT between 2007 and 2016 at five transplant centers. The median age at the time of allo-HCT was 8.4 years (range: 2.6 to 16.8 years). This cohort also included 5 patients with secondary HLH/MAS. Like our patient, all patients in this study received RIC with fludarabine- and alemtuzumab-based regimens [8]. At the last follow-up (median: 29 months; range: 2.8–96 months), of the 14 surviving patients, 11 were in CR, 1 was PR, and 2 had NR [8]. The authors acknowledged that continued long-term follow-up is required to assess rates of disease relapse and complications of the transplant.

Patients with sJIA are usually heavily pretreated with immunosuppressive agents to achieve disease control. Therefore, intense immune- and myeloablation are likely not required, and use of reduced-intensity conditioning regimens without the associated increased risk of rejection or mixed chimerism is justified in this population. Contrary to the report by Silva et al. [8] where none of the patients reported continuing anakinra treatment after HCT, we elected to administer anakinra through engraftment. This was done with an intent to suppress any potential flare of MAS in the peri-HCT period. It is also worth noting that due to the prolonged corticosteroid exposure, these patients are usually at a greater risk of secondary adrenal insufficiency, thereby necessitating a long and slow corticosteroid wean. At greater than 3 years after HCT, the patient has not had a recurrence of sJIA symptoms or MAS. Our report adds to the existing literature on Allo-HCT for patients with sJIA/MAS. Early intervention with RIC HCT in these patients should be considered, especially in the setting of an available match donor. To this end, prospective clinical trials incorporating RIC regimens and establishing transplant indication criteria are needed for this subset of patients.

## Data Availability

The additional data are available from the corresponding author upon request.

## Abbreviations

AI: Adrenal insufficiency
Allo-HCT: Allogeneic hematopoietic cell transplant
CR: Complete remission
GvHD: Graft-versus-host disease
HLH: Hemophagocytic lymphohistiocytosis
JIA: Juvenile idiopathic arthritis
MAS: Macrophage activation syndrome
MUD: Matched unrelated donor
NR: No response
PRs: Partial responders
RIC: Reduced-intensity conditioning
sJIA: Systemic juvenile idiopathic arthritis.
